# Incident Management Systems Are Essential for Effective Coordination of Large Disease Outbreaks: Perspectives from the Coordination of the Ebola Outbreak Response in Sierra Leone

**DOI:** 10.3389/fpubh.2016.00254

**Published:** 2016-11-21

**Authors:** Olushayo Oluseun Olu, Margaret Lamunu, Alexander Chimbaru, Ayotunde Adegboyega, Ishata Conteh, Ngoy Nsenga, Noah Sempiira, Kande-Bure Kamara, Foday Mohamed Dafae

**Affiliations:** ^1^World Health Organization (WHO), Kigali, Rwanda; ^2^World Health Organization (WHO), Geneva, Switzerland; ^3^World Health Organization (WHO), Freetown, Sierra Leone; ^4^World Health Organization (WHO) Intercountry Support Team for Eastern and Southern Africa, Harare, Zimbabwe; ^5^World Health Organization (WHO) Regional Office for Africa, Brazzaville, Congo; ^6^Ministry of Health and Sanitation, Freetown, Sierra Leone

**Keywords:** Ebola virus disease, outbreak response, coordination, incident management system, Sierra Leone

## Abstract

**Background:**

Response to the 2014–2015 Ebola virus disease (EVD) outbreak in Sierra Leone overwhelmed the national capacity to contain it and necessitated a massive international response and strong coordination platform. Consequently, the Sierra Leone Government, with support of the international humanitarian community, established and implemented various models for national coordination of the outbreak. In this article, we review the strengths and limitations of the EVD outbreak response coordination systems in Sierra Leone and propose recommendations for improving coordination of similar outbreaks in the future.

**Conclusion:**

There were two main frameworks used for the coordination of the outbreak; the Emergency Operation Center (EOC) and the National Ebola Response Center (NERC). We observed an improvement in outbreak coordination as the management mechanism evolved from the EOC to the NERC. Both coordination systems had their advantages and disadvantages; however, the NERC coordination mechanism appeared to be more robust. We identified challenges, such as competition and duplication of efforts between the numerous coordination groups, slow resource mobilization, inadequate capacity of NERC/EOC staff for health coordination, and an overtly centralized coordination and decision-making system as the main coordination challenges during the outbreak.

**Recommendations:**

We recommend the establishment of EOCs with simple incident management system-based coordination prior to outbreaks, strong government leadership, decentralization of coordination systems, and functions to the epicenter of outbreaks, with clear demarcation of roles and responsibilities between different levels, regular training of key coordination leaders, and better community participation as methods to improve coordination of future disease outbreaks.

## Introduction

With a total of 14,122 suspected, probable, and confirmed cases, and 3,955 deaths in all 14 health districts, the 2014–2015 Ebola virus disease (EVD) outbreak in Sierra Leone was the single largest in the history of the disease (1). The magnitude of the outbreak overwhelmed the national capacity to contain it and necessitated a massive international response, which included several dozen national and international partners with various mandates, interests, and comparative advantages (2, 3). During the initial response, similar to Guinea and Liberia, the other two principally affected countries, the effectiveness of the response was limited by several factors such as the magnitude of the outbreak, poor capacity for response and risk communication, ineffective coordination, weak health systems, and community resistance (4–6).

Involvement of numerous national and international partners in the outbreak response necessitated the establishment of a strong EVD outbreak response coordination platform. In response to this, the Sierra Leone Government, with support from the international humanitarian community, established and implemented various frameworks for the national coordination of the outbreak from July 2014 until November 2015 (7), when the outbreak was officially declared over. These frameworks aimed to address the different outbreak coordination needs, including command and control mechanisms for a multisectoral response; cross-border coordination of epidemic response interventions; development, implementation, and supervision of unified outbreak control guidelines at national and sub-national levels; effective communication between national and district response operations and between the different EVD response components, partners, and with the community.

Health coordination aims to ensure a coordinated and effective approach to emergency health program planning, implementation, supervision, and monitoring. It plays a critical role in ensuring available resources are effectively deployed and used, activities are well planned and synchronized and complement each other so that any gaps in the response operation are efficiently identified and resolved (8). Outbreak response requires strong and effective national leadership and coordination frameworks. This becomes more challenging and complicated during a vast outbreak like the EVD in West Africa that involved multiple stakeholders, some of which have limited technical knowledge and capabilities.

During our time supporting the outbreak response in Sierra Leone (please see the Section “Author Notes” for the roles of the authors in the coordination of the outbreak), we observed several positive features that facilitated effective coordination. Conversely, we also noted some barriers that may have delayed effective control of the outbreak. In this article, we identify the strengths and weaknesses of the EVD outbreak response coordination frameworks in Sierra Leone and propose a number of recommendations for improving the coordination of future outbreak responses.

## Organization of the National EVD Coordination System in Sierra Leone

There were two main periods and coordination frameworks for the EVD outbreak in Sierra Leone. From July to September 2014, an Emergency Operation Center (EOC) was used as the coordination model, whereas from October 2014 until the end of the outbreak, the Sierra Leone Government established a command and control style incident management system (IMS), called the National Ebola Response Center (NERC), to coordinate the outbreak.

### The EOC Coordination System

**Figure 1:**
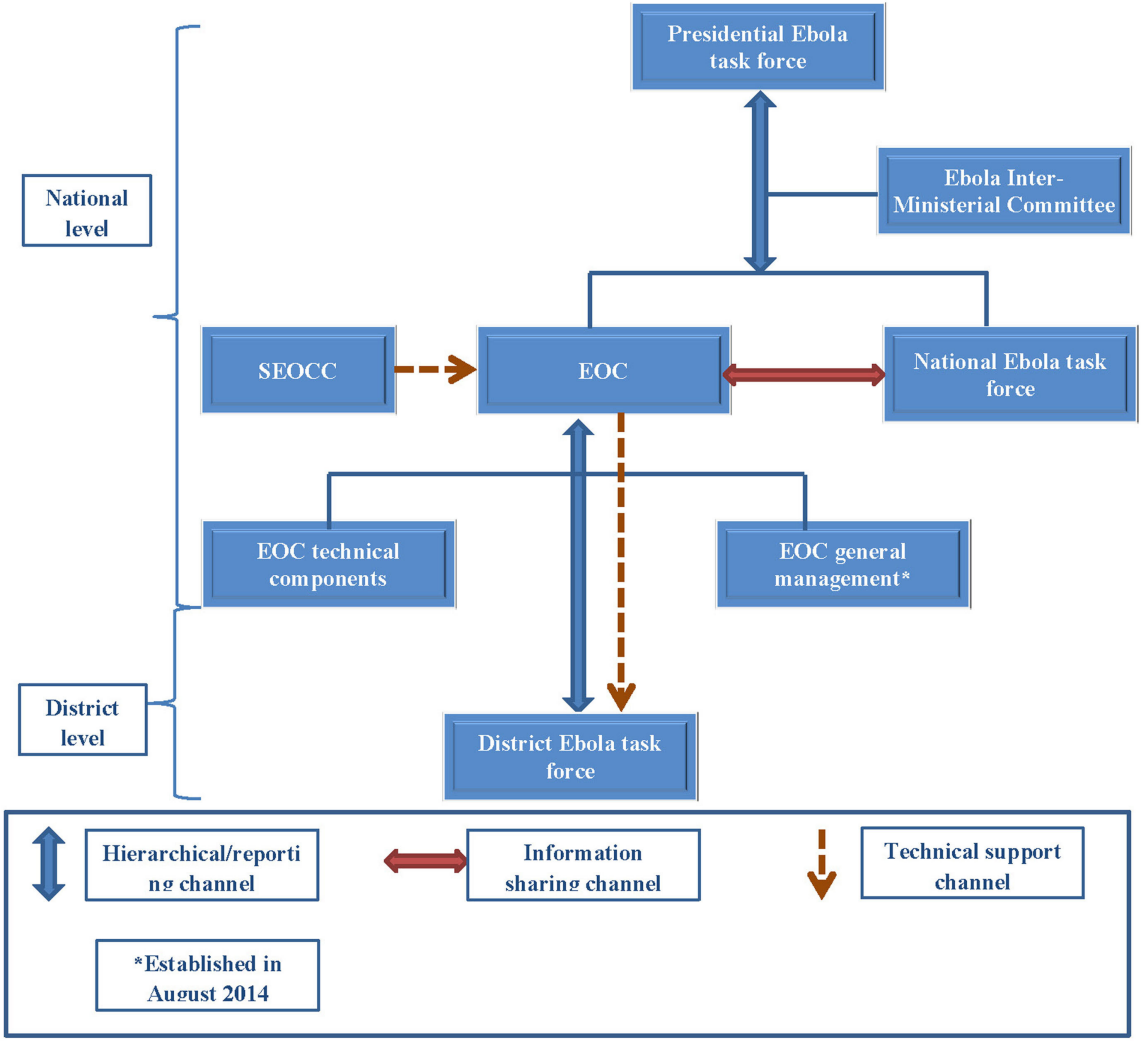
**Schematic diagram of the Ebola Operations Center (EOC), Sierra Leone: June–September, 2014**. Abbreviations: EOC, Ebola Operations Center; SEOCC, Sub-Regional Ebola Operation Coordinating Center.

Under the EOC coordination framework, three main EVD coordination groups, a national EOC, a Presidential task force, and a national EVD task force, were established at the national level (Figure 1). The national EOC was responsible for coordinating the operational and technical aspects of the outbreak response. This included operational planning, implementation, supervision, coordination, and monitoring and had six technical components; coordination, surveillance, and laboratory; case management; social mobilization; logistics; and psychosocial care/social protection. In addition to these components there was an Alert Call Center, which facilitated coordination of all community alerts. The EOC operated under the administrative authority of the Ministry of Health and Sanitation (MOHS) and was chaired by the Minister for Health/Chief Medical Officer (CMO) until the end of August 2014, when an Operations Coordinator was appointed by the Head of State. The CMO became solely responsible for coordination of the technical aspects, while overall administrative management of EOC was under the control of the Operations Coordinator. The national EOC reported to the Presidential taskforce through an inter-ministerial committee.

A Presidential EVD task force, chaired by the Head of State, provided overall policy, strategic, and political direction for the outbreak response. An inter-ministerial committee, which included relevant cabinet ministers, heads of relevant multilateral agencies such as the United Nations (UN) and international non-governmental organizations (NGOs) and bilateral partners such as US Centers for Disease Prevention and Control and Public Health England, oversaw implementation of the decisions of the Presidential task force. A national EVD task force was also established, with the aim of involving a broader spectrum of national, district, and community stakeholders in the EVD response. In reality, this task force, which only met on a few occasions, did not function effectively.

At the district level, the EOC coordination structure (district Ebola task forces) comprised five technical components; coordination; epidemiology/surveillance/laboratory; case management/infection control; social mobilization/psychosocial support; and logistics. The district task forces were chaired by the district council Chairmen, who organized daily meetings; each technical group also met on a daily basis. The coordination component included the leads for all the technical components, and discussions in these meetings were similar to those of the district task force meetings. At the supra-national level, a multiagency body, known as the Sub-regional Ebola Operations Coordinating Center (SEOCC), based in Conakry, Guinea, which coordinated the international and cross-border aspects of the outbreak and provided technical support to the national EOCs, was operational from June to October 2014.

### The NERC Coordination System

**Figure 2:**
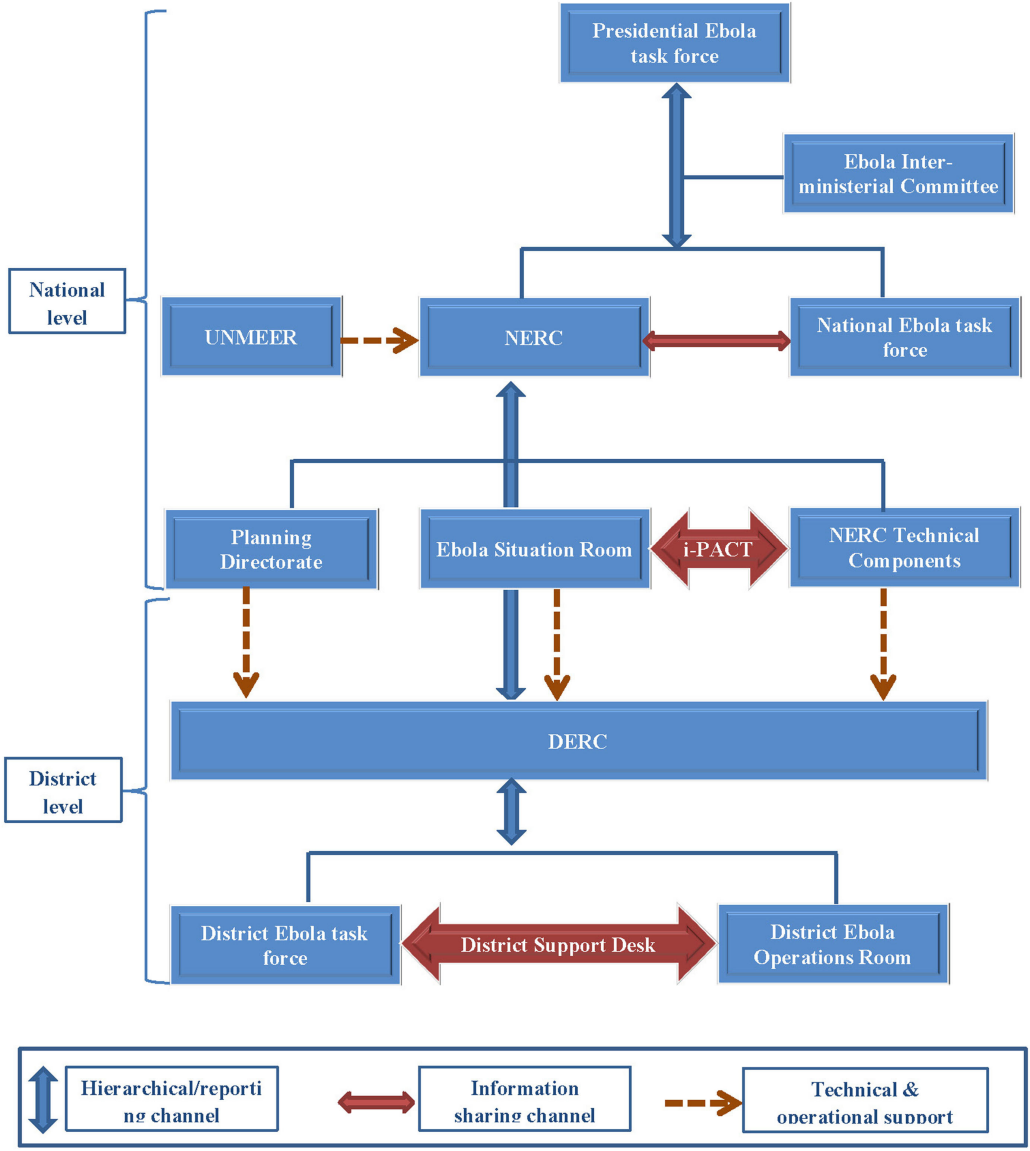
**Schematic diagram of the National Ebola Response Center (NERC), Sierra Leone: October 2014–November 2015**. Abbreviations: DERC, District Ebola Response Center; NERC, National Ebola Response Center; i-PACT, Inter-pillar Action and Coordination Team; UNMEER, United Nations Mission for Ebola Emergency Response.

The NERC, which succeeded the EOC in October 2014, was more sophisticated and incorporated a civil–military cooperative style of coordination, with deployment of Sierra Leonean and other foreign military personnel to support coordination and operation of the EVD response. It was chaired by a Chief Executive Officer who reported directly to the Head of State. Under this coordination framework, the operational and technical aspects of the outbreak response were clearly separated, with the establishment of a situation room that focused on monitoring the operational aspects of the outbreak, and the CMO leading the technical aspects, which were similar to the former EOC (Figure 2). The technical components continued to play their previous role in the outbreak response, although in a restructured form with seven components. The Presidential and national EVD task forces continued to operate without any changes. The NERC was housed in the UN Special Court, which had optimal space and equipment for its operations.

An Inter-pillar Action and Coordination Team (i-PACT) was subsequently established to strengthen leadership and bridge the communication gap between the two main NERC groups; the situation room and the technical components. Structures similar to the NERC, District Ebola Response Centers (DERCs), with district Ebola situation rooms and Ebola response components, each with a district coordinator, were established at the district level. In October 2014, the United Nations Mission for Ebola Emergency Response (UNMEER) replaced the SEOCC. National and district offices of UNMEER were established in the three principally affected countries in October 2014, each with its own coordinator.

## Key Limitations of the EVD Outbreak Coordination Systems in Sierra Leone

### General Observations

In general, we observed progressive improvements in the coordination of the outbreak (7). However, the coordination mechanisms remained centralized and top-heavy throughout. The EOC structure was simple and less labor-intensive compared to the NERC, with the main focus on technical issues and the development of standard operating procedures, with little emphasis and limited capacity to support the operational activities mainly due to lack of resources and technical know-how on emergency operations. This resulted in poor oversight, supportive supervision and monitoring, as well as inadequate links between the national and district coordination mechanisms. The EOC experienced bureaucratic decision-making, lack of funding, and low thresholds for the disbursement of available funds, which compromised its ability to effectively coordinate and respond to the outbreak. The most likely reason for this was the lack of executive power on decision-making, which also limited its ability to effectively coordinate with other sectors. Furthermore, combining the operational and technical aspects of the coordination efforts under one coordination structure proved to be challenging and often overwhelmed the capacity of the EOC. This was further compounded by inadequate infrastructure, including lack of office space, communication equipment, internet access, and limited staffing in the EOC. On the other hand, the NERC had far reaching decision-making authority due to its direct report to the Head of State. This, together with availability of required resources (both financial and human), adequate equipment, and space, made its operations more effective.

### Communication

At the initial implementation stages of the NERC system, there was a lack of clarity about the roles, responsibilities, and channels of communication between the different coordination groups. This was particularly evident with the District Health Management Teams (DHMT), which comprised the district EOCs and the newly established DERCs. The same situation was experienced at the national level, with the technical components operating independently from the operational components, without effective collaboration with the NERC (and the Ebola situation room).

Communication between the operational and technical components of the NERC was often suboptimal, at both district and national levels. This was mainly because of a lack of understanding of the new coordination mechanism, as no orientation was provided on the new mechanisms for civil–military cooperation. This often resulted in duplication of efforts and unnecessary competition between the two NERC groups (the operation room and technical components). This was reflected in the production of different technical epidemiological information by both groups.

Furthermore, there were at least three EVD coordinators at the district level (DERC coordinators, District Medical Officers, and UNMEER coordinators) leading to tension and conflict over control of the outbreak response. In districts such as Bombali, Port Loko, Kambia, and Western Area, foreign military personnel were also designated as coordinators, further compounding this conflict. Technical meetings were held with no clear mechanism for conveying recommendations to the main coordinating group. The i-PACT system was established to provide technical advice and improve communication between the two main coordination groups of the NERC at the national level; however, it achieved limited success. There was no direct funding to facilitate coordination between national and district level technical components.

### Community Engagement

The community was involved in the coordination of the response at the district level, through regular participation of the paramount Chiefs at daily DERC meetings. This was however, not sufficient to obtain the full cooperation of community members on response activities such as active case finding, contact tracing, burials, and social mobilization. It appeared that the inadequate cooperation was because of misconceptions and fear. One of which was the perception that the treatment centers represented “points of no return”; whoever was taken there was thought not likely to return to the community alive. This resulted in community resistance, which was a distinct feature of this outbreak (6, 9).

### EVD Response Coordination Meetings

During the initial stages of the outbreak, the management of some of the coordination meetings was poor; meetings were unnecessarily long, and some did not reach any concrete decisions. This was evidenced by poorly prepared minutes and the lack of clear mechanisms for follow-up on agreed action points. Consequently, there were often delays of several days to weeks before action points were implemented. This often resulted in apathetic officials, poor attendance, and repeated discussions of the same topics.

Many officials were members of multiple technical components or coordination groups, and the roles of other officials overlapped, resulting in the engagement of some of the key actors in frequent and prolonged meetings. For instance, the district Ebola task forces and their technical coordination component essentially included the same people and discussed the same issues. Slight reductions in these challenges were noted under the NERC system, which streamlined the number, participation in, and duration of coordination meetings. For example, the national Ebola situation room met twice a day for a maximum of 1 h. Conversely, this led to concerns from some partners about the limited time for in-depth discussions of critical issues.

### EVD Information Management

Our observations highlighted multiple EVD information sharing channels and situation reports, each conveying different information and epidemiological data. This often fueled disagreements between partners resulting in poor use of the available data to guide the ongoing response. For instance, many stakeholders had different data collection guidelines and reporting formats, which challenged data aggregation at the national level. This resulted in poor coordination of the EVD data collection system and several versions of the national EVD database, each of which had its own limitations (10). At the later stages of the outbreak, a district support and assurance desk was established within the NERC to bridge the information gap between the districts and the national level and consolidate NERC support to the districts.

### EVD Resource Mobilization and Disbursement

We observed that weak coordination of the mobilization, and deployment of the resources required for the EVD outbreak response was one of the most critical challenges for effective control of the outbreak, particularly during the early phases. The logistical components were unable to estimate or quantify available logistics, supplies, and essential medicines. This led to inaccurate forecasts, which often resulted in insufficient supplies, as most medical supplies were imported. This was largely because of multiple supply chain systems, many of which were not captured in the national EVD inventory systems. The EVD resources, which were channeled through the EOC and NERC systems, often arrived late at the district level because of the bureaucratic financial and logistical management systems of both the EOC and NERC.

Furthermore, the EVD coordination hubs (and associated resources) were often not located in appropriate places. This resulted in a breakdown in communication between the field teams and coordination hubs and delays in receiving the required support to the field teams. For example in Koinadugu district, although the epicenter of the outbreak was in Neine, a remote chiefdom at least 4–5 h drive from the district headquarters, the outbreak coordination mechanisms and resources were predominantly located at the district headquarters. This severely limited the initial response to the outbreak in that area.

### Supervision, Monitoring, and Evaluation of EVD Outbreak Response Interventions

The supervision, monitoring, and evaluation component of the EVD coordination system was observed to be weak, late into the outbreak response. There was no clear system for ensuring support supervision from national to district levels and from district to ward levels, to ensure that corrective action was taken when required. Thus it was difficult to ensure the quality of interventions and to rapidly identify gaps in the response effort. From July 2015, the NERC, supported by partners, established a critical monitoring team to monitor the quality of the response in districts reporting cases, as well as the preparedness level in districts that were not reporting.

## Conclusion

Coordination of outbreak response efforts plays a part in breaking the chain of transmission but will not ultimately control it single-handedly. Nevertheless, effective coordination of disease outbreak response should be an integral and critical component of overall outbreak control interventions (11). Effective coordination of outbreaks, such as the 2014/15 EVD outbreak, requires strong government leadership and ownership (12), a common vision, good management processes, and a shared strategy and priorities between all involved stakeholders.

Coordination practices during the EVD outbreak highlighted improvement in outbreak coordination, as the mechanism evolved from the EOC to the NERC (7). Our observations indicated that both coordination systems had some similarities, advantages, and limitations. However, we believe that the NERC coordination mechanism was more robust as it provided a more effective platform for coordination of the outbreak and significantly contributed to its eventual control. The successes of the NERC system could be attributed to its executive powers for decision-making, human resource deployment, and fund disbursement. Its incident management style ensured that agreed actions were followed up in a timely manner, and the decision-making process was much more efficient (13). Furthermore, the separation of the operational and technical components of the response coordination under the NERC system ensured a more streamlined approach to coordination, which enabled the public health experts to focus on their technical work.

We observed several pitfalls, common to both coordination frameworks (EOC and NERC), which should be avoided during future outbreaks. These included competition and duplication of efforts between the numerous coordination groups; poor communication and relationships between the national and district coordination groups; weak and bureaucratic response components; and inadequate logistics and supplies. Other challenges include slow resource mobilization and disbursement because of bureaucratic systems; inadequate capacity of NERC/EOC staff for health coordination; overtly centralized and top-heavy coordination; and a decision-making system that concentrated on Freetown and district headquarters. Our observations also highlighted that although UNMEER was established to enhance outbreak coordination, its impact was limited (6) because of its lack of adequate technical capacity and expertise about EVD, inadequate logistic capacity, and its duplication of some of the NERC and DERC functions. Furthermore, the body duplicated the functions of the United Nations Office for Coordination of Humanitarian Affairs, which is an existing international organization tasked with humanitarian coordination (3, 6). This meant the humanitarian cluster coordination system (14), which is the internationally accepted framework for coordinating humanitarian crises, was never formally activated during this outbreak. Our observations compare favorably to those of other studies, which reviewed coordination of emergency response interventions (3, 6, 7, 11, 15).

We believe that early implementation of an IMS coordination system, such as the NERC, which is based on humanitarian cluster coordination principles, combined with a command and control style of coordination would have provided opportunities for better coordination of the outbreak response at the initial stages (16–18). This belief is premised on the successful use of IMS to manage the EVD outbreaks in Guinea, Liberia, and Nigeria with good results (7, 11, 16, 17). In Liberia, the IMS facilitated well organized outbreak management, clear chain of command, accountability, and timely follow up of actions (11, 16); similar benefits were also observed in Nigeria (17). However, the IMS is not without its pitfalls; its implementation in Sierra Leone was labor and capital intensive and was initially poorly understood by the national authorities and partners. Thus, careful planning and roll out of IMS is required to reduce duplication and transaction costs and to ensure better civil–military cooperation and coordination.

## Recommendations

Based on our observations, we propose five recommendations to improve a coordinated response needed to control this type of disease outbreak in the future.

First, all countries should establish EOCs, with simple IMS-based coordination mechanisms as part of outbreak preparedness and response efforts. Such centers should be led and maintained by the government (12, 19) to ensure sustainability and have executive decision-making powers where necessary. The centers should ensure clear separation between the technical and operational aspects of the outbreak coordination, facilitate adequate links between the various administrative levels, and ensure regular information sharing with streamlined outbreak information dissemination channels to guarantee a unified system for information sharing. Furthermore, the quality of coordination meetings should be improved by good minute recording and putting in place appropriate mechanisms for the follow-up of agreed action points. A comprehensive national inventory system to ensure coordinated resource mobilization and deployment is also imperative.

Second, decentralization of coordination systems and functions to the epicenter of outbreaks, with a clear demarcation of roles and responsibilities between the national, districts, and local levels should be encouraged. The national level should focus on technical guidance, policy and strategy development, resource mobilization, and technical oversight, while the district should be empowered to conduct the day-to-day coordination and operation of the outbreak response.

Third, on-the-job training of all key coordination leaders to ensure better understanding of the coordination mechanism and enhance their coordination capacity is critical to enhance effective coordination. The content of such training should include general principles of health coordination, management of coordination meetings, strategies for identification and filling of critical gaps in the response, communication, conflict management, and inventory/logistics management skills.

Fourth, better community engagement and participation in the coordination of future EVD outbreaks control is imperative. Modalities for ensuring better engagement of the community, such as conducting ongoing anthropological studies to better understand the community context and cultural norms should be prioritized.

Finally, operational researches and monitoring and evaluation systems are needed to better understand outbreak coordination dynamics and to assess the effectiveness of health coordination mechanisms during disease outbreak responses in resource constrained settings, such as in the countries affected by Ebola.

## Author Notes

OO, ML, and NN were WHO Ebola response national technical coordinators in Sierra Leone at various times. In this capacity, they represented WHO in the EOC/NERC and were actively involved in the operations of these bodies. AC was WHO liaison officer to the EOC/NERC and was involved in the establishment and day-to-day management of the bodies. AA, IC, and K-BK were WHO district Ebola response coordinators in various districts. In this capacity, they represented WHO in the district EOC/DERC and were actively involved in the coordination of the outbreak at the district level. NS was WHO data management support to Western Area district, one of the epicenters of the outbreak, and participated actively in the coordination of the surveillance and epidemiology component. FD is a staff of MOHS Sierra Leone who was based in the EOC/NERC and actively participated in their establishment and day-to-day management.

## Author Contributions

OO, ML, AC, AA, and IC conceived and coordinated the study. OO, ML, AC, IC, AA, NN, NS, K-BK, and FD drafted the manuscript. All authors read and provided significant inputs into the final draft of the manuscript, agreed to be accountable for all aspects of the work, and approved the final draft of the manuscript for publication.

## Conflict of Interest Statement

The authors declare that the research was conducted in the absence of any commercial or financial relationships that could be construed as a potential conflict of interest. The handling Editor declared a past coauthorship with the authors and states that the process nevertheless met the standards of a fair and objective review.

## Abbreviations

CMO, chief medical officer; DERC, District Ebola Response Center; DHMT, District Health Management Team; EOC, Ebola Operations Center; IMS, incident management system; i-PACT, Inter-pillar Action and Coordination Team; MOHS, Ministry of Health and Sanitation; NERC, National Ebola Response Center; NGO, Non-Governmental Organization; SEOCC, Sub-Regional Ebola Operation Coordinating Center; UN, United Nations; UNMEER, United Nations Mission for Ebola Emergency Response.
